# Perampanel Reduces Hyperthermia-Induced Seizures in Dravet Syndrome Mouse Model

**DOI:** 10.3389/fphar.2021.682767

**Published:** 2021-07-14

**Authors:** Shih-Yin Ho, Li Lin, I-Chun Chen, Che-Wen Tsai, Fang-Chia Chang, Horng-Huei Liou

**Affiliations:** ^1^ Department of Neurology, National Taiwan University Hospital and National Taiwan University College of Medicine, Taipei, Taiwan; ^2^ Department and Graduate Institute of Pharmacology, College of Medicine, National Taiwan University, Taipei, Taiwan; ^3^ Department of Veterinary Medicine, School of Veterinary Medicine, National Taiwan University, Taipei, Taiwan; ^4^ National Taiwan University Hospital Yunlin Branch, Douliu, Taiwan

**Keywords:** epilepsy, SCN1A, stiripentol, valproic acid, phenytoin, NBQX

## Abstract

Treatment options for Dravet syndrome are limited. The aim of this study was to evaluate the antiepileptic effect of the AMPA receptor antagonist perampanel (PER) on a mouse model of Dravet syndrome (*Scn1a* ^*E1099X/+*^). We report here that the PER (2 mg/kg) treatment inhibited the spontaneous recurrent seizures and attenuated epileptic activity in *Scn1a* ^*E1099X/+*^ mice. In the hyperthermia-induced seizure experiment, PER clearly increased temperature tolerance and significantly ameliorated seizure frequency and discharge duration. PER also demonstrated antiepileptic effects in a cross-over study and a synergistic effect for attenuating heat-induced seizure when given in combination with stiripentol or valproic acid. The results showed that PER effectively decreased the occurrence of spontaneous recurrent seizures and showed significant therapeutic potential for hyperthermia-induced seizures with regard to both susceptibility and severity in a Dravet syndrome mouse model. Potential therapeutic effects of PER for treatment of Dravet syndrome were demonstrated.

## Introduction

Dravet syndrome, also known as severe myoclonic epilepsy in infancy, is a catastrophic and drug-resistant epileptic encephalopathy, with an incidence of about 1 per 20,000 to 40,000 (Wu et al., 2015). Dravet syndrome typically starts during the first year of life, and seizure symptoms often appear during high temperatures such as hot bathing or fever (Dravet and Oguni, 2013). In addition to hyperthermia-induced epilepsy, patients with Dravet syndrome also experience spontaneous recurrent seizures. Several types of seizures including tonic–clonic, myoclonic, absence, partial, and atonic are identified in Dravet syndrome patients (Aras et al., 2015).

Approximately 70–80% of Dravet syndrome patients have loss-of-function mutations in the sodium voltage-gated channel alpha subunit 1 (*SCN1A*) gene (Marini et al., 2011). The protein encoded by this gene is voltage-gated sodium channel Na_v_1.1 which is predominantly expressed on the initial axon segment of the parvalbumin-positive gamma-aminobutyric acid (GABA) interneuron (Ogiwara et al., 2007). This genetic defect results in decreasing exocytosis of GABA from the axon terminal. Previous work in our and other laboratories supports the hypothesis that the mechanism of epileptogenesis in Dravet syndrome is insufficient GABA release leading to brain hyperexcitability (Yu et al., 2006; Ogiwara et al., 2007; Tsai et al., 2015).

Medical options for Dravet syndrome are quite limited. Current first-line treatment drugs for Dravet syndrome are valproic acid, clobazam, and stiripentol (Wirrell, 2016). These drugs have been shown to have the ability to promote inhibitory neurotransmission by inhibiting GABA metabolism, reducing GABA reuptake, or promoting improvement in the opening of GABA receptors. Several types of sodium channel blockers such as phenytoin, carbamazepine, and lamotrigine have been shown to exacerbate the seizure-related symptoms of Dravet syndrome and should be avoided (Mantegazza et al., 2010). To date, only cannabidiol (proposed to enhance GABAergic inhibitory signaling by blocking GPR55) and stiripentol have been approved for the treatment of Dravet syndrome by the US Food and Drug Administration (Wirrell and Nabbout, 2019). Therefore, there is an urgent need to search for a new target for the treatment of Dravet syndrome. Recently, some clinical studies demonstrated that PER treatment reduced seizure frequency and may be effective in patients with Dravet syndrome (Lin et al., 2018; Yoshitomi et al., 2019).

PER is the first and so far the only α-amino-3-hydroxy-5-methyl-4-isoxazolepropionic acid (AMPA) glutamate receptor inhibitor in clinical use. PER has been approved for add-on treatment of partial onset seizure in patients aged >12 years (Rosenfeld et al., 2015; Greenwood and Valdes, 2016). Compared with some commonly used clinical antiepileptic drugs (AEDs) that may cause serious severe and fatal side effects, such as Stevens–Johnson syndrome (Frey et al., 2017), PER is rather mild with regard to side effects. The most common side effects are dizziness, ataxia, headache, and somnolence (Rugg-Gunn, 2014). In addition to its therapeutic effect on partial onset seizure, PER has also demonstrated broad-spectrum antiseizure effects in many animal epilepsy models, such as the maximal electroshock mouse model, audiogenic seizure mouse model, pentylenetetrazol (PTZ) mouse model, 6-Hz mouse model, amygdala kindling rat model, and lithium–pilocarpine rat model (Hanada et al., 2011; Mohammad et al., 2019). Although PER is approved for treatment of partial onset seizure and has been found to have antiepileptic effects in many animal models, the efficacy of PER monotherapy and combination therapy for Dravet syndrome still needs to be determined. The purpose of this study was to examine the therapeutic effect of PER in single-drug and multiple-drug applications on a Dravet syndrome mouse model.

## Materials and Methods

### Animals

A Dravet syndrome mouse model (C57BL6/J x 129/SvCrl genetic background) with loss-of-function mutation E1099X in the *Scn1a* gene, which encodes voltage-gated sodium channel Na_v_1.1, was created (Tsai et al., 2015). This mouse model was created by using the knock-in method to convert the GAG codon in the target position of the *Scn1a* gene into TAG. TAG is a stop codon which prevents the Na_v_1.1 protein from being completely translated, thereby causing functional defects in the sodium channel. When mice with *Scn1a* haploinsufficiency are bred with each other, their progeny will produce three genotypes: *Scn1a* ^*+/+*^ (wild-type mice), *Scn1a* ^*E1099X/+*^(Dravet syndrome mice), and *Scn1a* ^*E1099X/E1099X*^. The body weight and activity of the *Scn1a* ^*E1099X/E1099X*^ genotype during development are significantly abnormal compared with those of normal mice, and most cannot survive for more than 9 days. Because of this severe condition, they were not used for this study. All mice were housed separately in individual recording cages in the isolation room, in which the temperature was maintained at 22 ± 2°C, the light/dark rhythm was controlled in a 12/12 h cycle, and there was unlimited food and water. All procedures performed in this study were approved by the National Taiwan University Animal Care and Use Committee.

### Electroencephalograms

**FIGURE 1:**
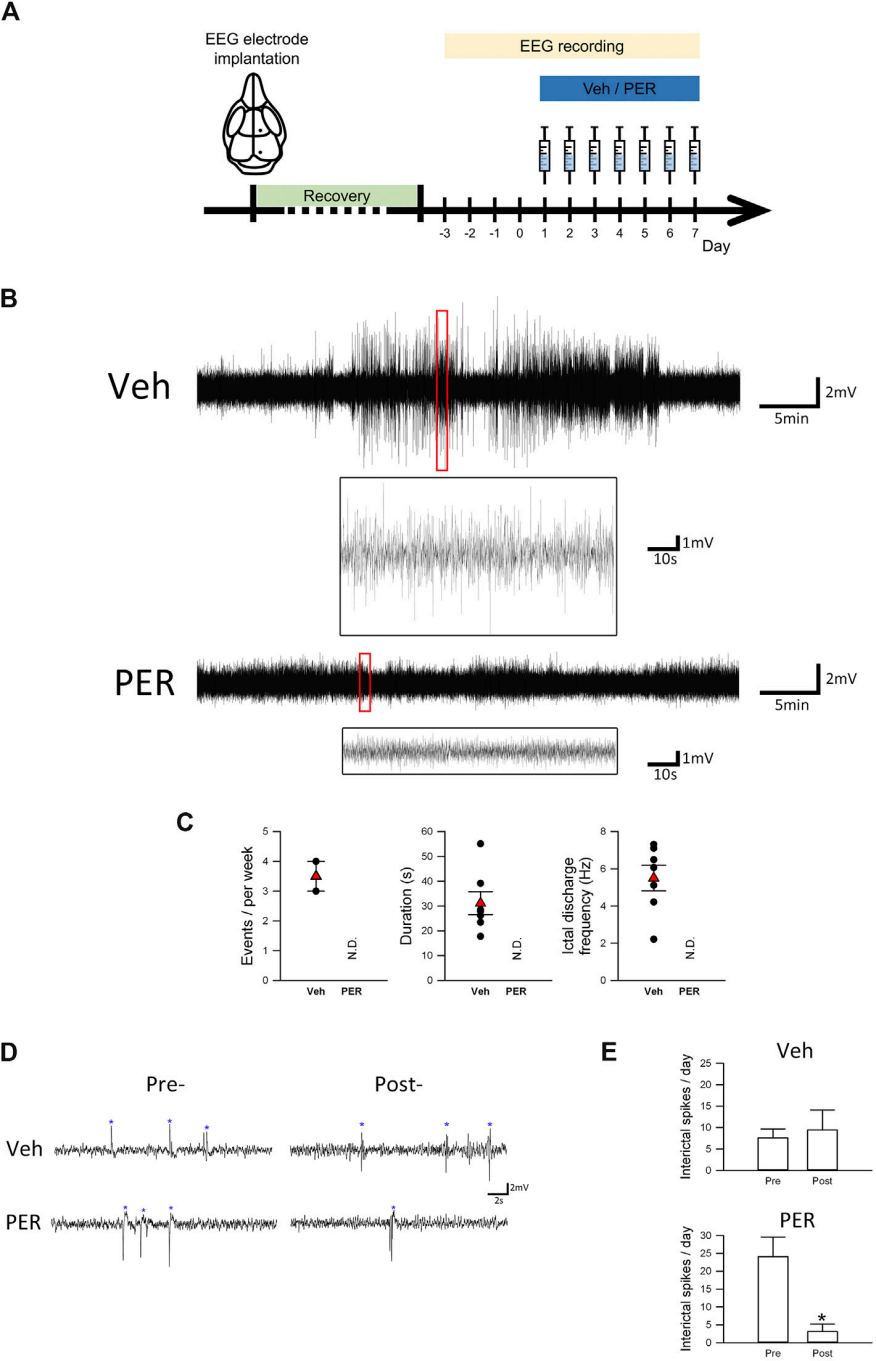
Perampanel suppressed seizure activity in *Scn1a* ^*E1099X/+*^ mice with spontaneous recurrent seizures. **(A)** Schematic illustration of the experimental protocols (each illustration is an original drawing made by the authors). **(B)** Representative EEG trace (2.2–7.0 Hz) of one spontaneous seizure event in vehicle and PER treatment groups (*n* = 3 per group). **(C)** Quantitative results of the ictal discharge event during EEG recording, including seizure duration and frequency in vehicle and PER treatment groups (*n* = 3 per group). N.D., not determined. **(D)** Interictal spikes (indicated by asterisks) are illustrated. Interictal spikes in the PER treatment group (*n* = 3 per group) were significantly decreased during a period of seven-day administration in comparison with the vehicle group (*n* = 3 per group). **(E)** Quantitative results of interictal spikes from **(D)**. *p < 0.05, Student’s paired *t*-test.

The procedures for electroencephalography (EEG) were modified from those used by Yi et al. (2015). Briefly, mice were anesthetized by intraperitoneal injection with 50 mg/kg tiletamine hydrochloride and zolazepam hydrochloride (Zoletil®, Virbac, Carros, France), and surgery was performed using a stereotaxic instrument. Simultaneously, EEG electrodes were implanted under the skull and over the cortex (1.7 mm lateral to the midline and 1.5 mm anterior to bregma) and at the cerebellum (1.2 mm lateral to the midline and 1.5 mm anterior to lambda) as a reference electrode (Figure 1A). The EEG signal was amplified by an amplifier (model V75-01; Colbourn Instruments, Lehigh Valley, PA, United States), bandpass filtered between 0.1 and 40 Hz, and subjected to analog-to-digital conversion with 16-bit precision at a sampling rate of 128 Hz (NI PCI-6033E; National Instruments, Austin, TX, United States). To examine the proportion of mice with spontaneous recurrent seizures, the entire 24-h recording was inspected visually for each mouse. For quantification of spontaneous recurrent seizures’ occurrence, the epileptic spikes were visually analyzed by AxoScope 10 software (Molecular Devices, Sunnyvale, CA, United States). The epileptiform EEG patterns were defined as the epileptic discharges with amplitude ≥ 2 mV, duration ≥ 5 s, and frequency ≥ 2 Hz. Meanwhile, the interictal spikes were recognized by the following parameters: intermittent events with large amplitudes (≥6 times the standard deviation of background signals), simple or complex waveforms, and a duration of 30–250 ms. Interictal spikes were collected ≥30 min after a preceding ictal event to avoid the influences of ictal discharges on subsequent interictal spikes.

### Heat-Induced Seizures

The procedure of heat-induced seizures was performed as described by Oakley et al. (2009). Briefly, mice aged 4–6 weeks were placed in a small cage and heated using a heat lamp. Rectal temperature was continuously measured with a temperature probe during this hyperthermia-induced seizure experiment. The core body temperature was then elevated approximately 0.5°C every 2 min until either seizure occurred or 42.5°C was reached. The epileptiform EEG patterns were the same as mentioned above. Seizure severity was quantified by using a modified Racine scale (Luttjohann et al., 2009): (0) no change in behavior; (1) sudden behavioral arrest, motionless staring (with orofacial automatism); (2) head nodding; (3) forelimb clonus with lordotic posture; (4) forelimb clonus, with rearing and falling; and (5) generalized tonic–clonic activity with loss of postural tone, often resulting in death, wild jumping.

### Drug Administration

Antiepileptic drug monotherapy has been thought to be the first-line treatment for newly diagnosed epileptic patients (Zeng et al., 2015). Combination of AED therapy will be considered, when monotherapy does not work for the patient to achieve seizure control. PER is mainly bound with plasma protein (95%) and absorbed completely and rapidly with average 105 h half-life (Zeng et al., 2015). The PER (1–2 mg/kg) dosage used in the present study is adapted from the previous report (Hanada et al., 2014). Drugs including phenytoin (100 mg/kg), valproic acid (120 mg/kg), stiripentol (300 mg/kg), and NBQX (30 mg/kg) were also used in the study. All drugs were dissolved in the vehicle solution (distilled water, dimethyl sulfoxide (DMSO), polyethylene glycol 300, in a 1:1:1 ratio) as previously described (Hanada et al., 2014). The drugs were administered by intraperitoneal injection or given orally (*via* oral gavage) based on the experimental purpose. In the cross-over experiments, the mice were randomly divided into two groups. The injection strategies in Group1/Group2 mice were as follows: Group1, Veh (first week)–PER (second week)–Veh (third week), and Group2, PER (first week)–Veh (second week)–PER (third week). The hyperthermia-induced seizures were performed 30 min after drug treatment.

### Statistical Analysis

Statistical analysis was done using GraphPad Prism version 7 software (La Jolla, CA, United States). Results were examined by Student’s *t*-test for two-group comparisons, and one-way analysis of variance (ANOVA) followed by Tukey’s post hoc test was used for multiple-group comparisons. The Mann–Whitney and Kruskal–Wallis tests were used when comparing two groups or greater numbers of variables of Racine score results, respectively. In the hyperthermia-induced seizure experiments, logrank Mantel–Cox tests followed by Bonferroni’s post hoc comparison tests were performed. Data are presented as mean ± SEM. A *p* value < 0.05 is considered statistically significant.

## Results

### Effect of PER on Seizure Activity in *Scn1a* ^*E1099X/+*^ Mice With Spontaneous Recurrent Seizures

Figure 1A shows that, after EEG surgery was performed on mice aged 4–6 weeks, it was followed by 3–5 days of recovery. After 24-hour EEG recording at baseline for 4 days, vehicle or PER (2 mg/kg) was given daily for seven consecutive days. In addition to normal baseline recordings, several ictal discharge events were observed (Figure 1B). Statistical analysis showed that, during the 7 days of vehicle administration, 2/3 (66.7%) of mice had spontaneous recurrent seizure. An average of 3.33 ictal discharge events were observed per mouse during one week. However, 0/3 (0%) of the Dravet syndrome mice given PER had spontaneous recurrent seizure. Further analysis of the characteristics of these ictal discharge events showed that the average duration of each event was 31.1 ± 4.7 s and the average ictal discharge frequency of each event was 5.5 ± 0.7 Hz (Figure 1C).

In addition to ictal discharge events, Dravet syndrome mice may also produce interictal spikes due to excessive discharge of neurons (Figure 1D). In the vehicle group, we found no significant difference in interictal spikes number in Dravet syndrome mice before and after vehicle treatment (pretreatment, 7.6 ± 2.1 spikes/day; posttreatment, 9.4 ± 4.7 spikes/day). However, in the PER group, the number of spikes was significantly decreased during the total of seven-day recording (3.1 ± 2.1 spikes/day) compared with the pretreatment period (24.1 ± 5.5 spikes/day, *t*_(2)_ = 3.572, *p* = 0.0233, Figure 1E). Notably, we found the number of interictal spikes in the PER group before the treatment was obviously higher. This discrepancy may be caused by the different cohorts of mice used for the tests. These results showed that PER can prevent spontaneous recurrent seizure events and decrease interictal pattern occurrence in Dravet syndrome mice.

### Assessment of Efficacy of PER in *Scn1a* ^*E1099X/+*^ Hyperthermia-Induced Seizure Mice

**FIGURE 2:**
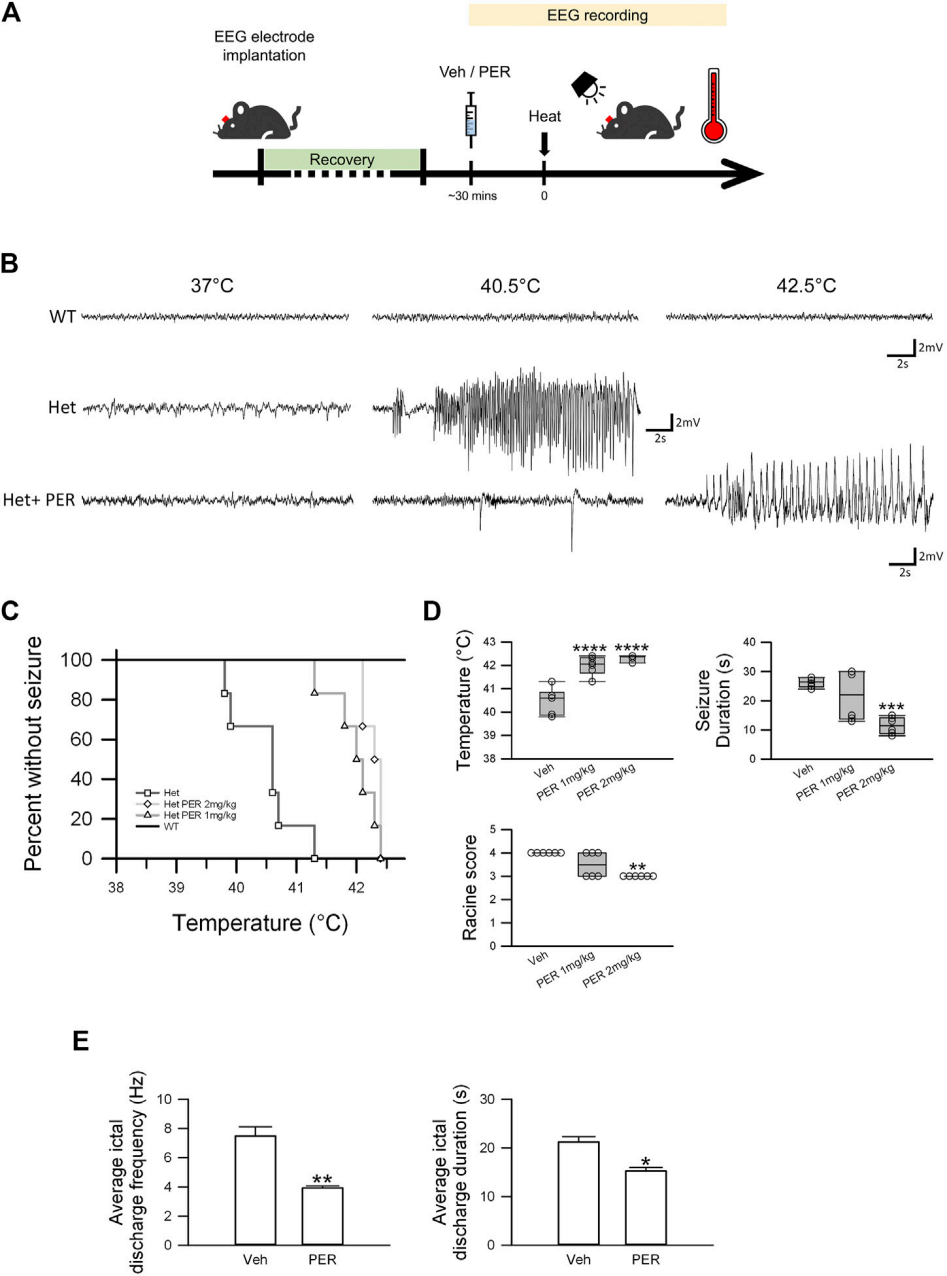
Efficacy of PER monotherapy in hyperthermia-induced seizure *Scn1a* ^*E1099X/+*^ mice. **(A)** Schematic illustrations of the protocol of the hyperthermia-induced seizure experiments. **(B)** Representative EEG trace (3.9–7.5 Hz) of a spontaneous seizure in vehicle and PER treatment groups (*n* = 6 per group) under different hyperthermia. **(C)** Percentage of mice remaining free of seizure plotted against core temperature. *p* = *0.012*, Het vs. Het PER 1 mg/kg; *p* = *0.0005*, Het vs. Het PER 2 mg/kg. *p*-Values were determined by logrank Mantel–Cox tests followed by Bonferroni’s post hoc comparison tests with *n* = 6 per treatment group. **(D)** Quantitative results of temperature, seizure duration, and Racine score in the vehicle group, 1 mg/kg PER treatment group, and 2 mg/kg PER treatment group (*n* = 6 per group). ***p < 0.001, vehicle vs. Het PER 2 mg/kg in seizure duration; ****p < 0.0001, vehicle vs. Het PER 1 mg/kg and vehicle vs. Het PER 2 mg/kg in temperature. One-way ANOVA followed by Tukey’s multiple comparisons post hoc analysis. **p < 0.01, vehicle vs. Het PER 2 mg/kg in Racine score. The Kruskal–Wallis test. **(E)** The ictal discharge frequency and duration of the vehicle group (*n* = 3) and 2 mg/kg PER treatment group (*n* = 3) are illustrated. *p < 0.05, vehicle vs. Het PER 2 mg/kg in duration; **p < 0.01, vehicle vs. Het PER 2 mg/kg in frequency. Student’s unpaired *t*-test.

Hyperthermia-induced seizure experiments were performed on the wild-type or Dravet syndrome mice 30 min after vehicle or PER administration, and EEG recording was carried out (Figure 2A). In the wild-type mice, no ictal discharge events were observed during the hyperthermia-induced seizure experiments from 37 to 42.5°C (Figure 2B). But in Dravet syndrome mice, all animals had induced seizure before the temperature reached 41.5°C (Figure 2C). The average induced temperature was 40.5 ± 0.2°C. With regard to induced seizure severity, the average seizure duration was 26.3 ± 0.7 s, and the Racine score was 4 (Figure 2D). In the PER group (1 mg/kg), all mice had induced seizure when the temperature ranged from 41 to 42.5°C. The average temperature for seizure induction was significantly raised to 42.0 ± 0.2°C which was significantly higher compared to that in the vehicle group (F_(2,15)_ = 34.233; *p* = < 0.0001, Figure 2D). But seizure severity was not significantly changed. The average seizure duration was 21.8 ± 3.5 s, and the Racine score was 3.5 (Figure 2D, PER 1 mg/kg). In the PER 2 mg/kg group, all mice had induced seizure from 42 to 42.5°C. The average temperature for induced seizure was significantly raised to 42.3 ± 0.1°C compared with that in the vehicle group (F_(2,15)_ = 34.233; *p* = < 0.0001). Furthermore, the average seizure duration was significantly decreased to 11.5 ± 1.2 s compared with that in the vehicle group (F_(2,15)_ = 12.233; *p* = 0.0006), and the Racine score was reduced to 3 (Figure 2C). In EEG studies, PER (2 mg/kg) administration also reduced seizure severity (Figure 2E). The average ictal discharge frequency was significantly decreased to 3.9 ± 0.1 Hz compared with that in the vehicle group (7.5 ± 0.6 Hz, *t*_(4)_ = 5.427, *p* = 0.0056), the average ictal discharge duration was significantly decreased to 15.3 ± 0.7 s compared with that in the vehicle group (21.2 ± 1.1 s, *t*_(4)_ = 4.582; *p* = 0.0102), and the Racine score was decreased to 3 (Figure 2D). These results showed that PER had significant efficacy with regard to decreasing hyperthermia-induced seizure susceptibility and severity.

### Perampanel Demonstrated Antiepileptic Effects in *Scn1a* ^*E1099X/+*^ Hyperthermia-Induced Seizure Mice

**FIGURE 3:**
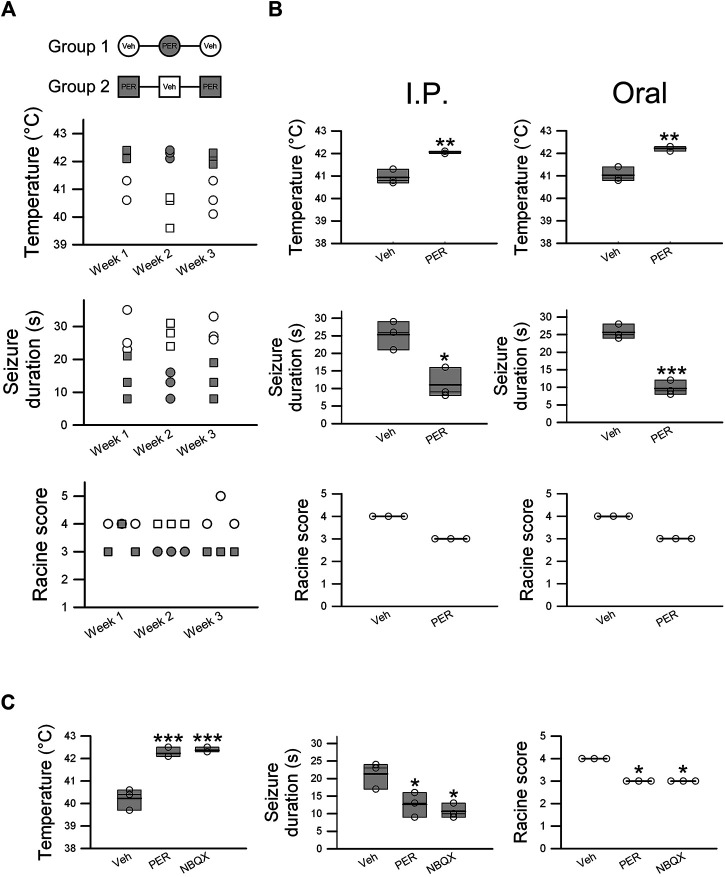
Perampanel exhibits effective antiepileptic activity in hyperthermia-induced seizures in *Scn1a* ^*E1099X/+*^ mice. Cross-over experiments were designed. **(A)** Mice were divided into two groups (Group1 and Group2, *n* = 3 per group), and temperature change, seizure duration, and Racine score were recorded for 3 weeks. Empty circles and squares represent the vehicle control group, and shaded circles and squares represent mice treated with PER 2 mg/kg. **(B)** Oral administration and intraperitoneal injection of PER 2 mg/kg were evaluated, and temperature change, seizure duration, and Racine score were determined in the vehicle group (*n* = 3) and 2 mg/kg PER treatment group (*n* = 3). *p < 0.05, **p < 0.01, and ***p < 0.001: vehicle vs. Het PER 2 mg/kg. Student’s unpaired *t*-test in temperature change and seizure duration. **(C)** NBQX (30 mg/kg), an AMPA receptor antagonist, was utilized for comparison of effectiveness with PER (2 mg/kg) as a positive control. The results of temperature change, seizure duration, and Racine score with PER (*n* = 3) and NBQX (*n* = 3) were similar. *p < 0.05, ***p < 0.001: vehicle vs. Het PER 2 mg/kg and vehicle vs. NBQX 30 mg/kg. One-way ANOVA followed by Tukey’s multiple comparisons post hoc analysis in temperature and seizure duration. The Kruskal–Wallis test in Racine score.

A cross-over study was also carried out. Hyperthermia-induced seizure was performed for three consecutive weeks. Hyperthermia-induced seizure susceptibility and severity were decreased every week PER (2 mg/kg) was administered, but this effect was not observed every week vehicle was administered (Figure 3A). These results proved that the therapeutic effects are not caused by individual differences. Oral PER, which is how PER is mostly administered in clinical practice, was given at the same dose as was given by intraperitoneal injection (2 mg/kg), and the same hyperthermia-induced seizure experiment was performed. The results showed that oral PER had almost the same therapeutic effects on both seizure susceptibility and severity as intraperitoneal injection of PER (Figure 3B), which showed that PER had excellent oral bioavailability. To try to further understand the antiepileptic mechanism, NBQX (30 mg/kg), an AMPA receptor antagonist, was used as a positive control for comparison with PER (2 mg/kg). The results showed that differences between induced temperature in the PER group (42.2 ± 0.1°C) and the NBQX group (42.4 ± 0.1°C), seizure duration in the PER group (12.7 ± 2.0 s) and the NBQX group (10.7 ± 1.2 s) group, and Racine score in the PER group (*n* = 3) and the NBQX group (*n* = 3) were not statistically significant (Figure 3C). Thus, most likely, PER exerts antiepileptic effects by the inhibition of AMPA receptors.

### Efficacy of Monotherapy of Different Antiepileptic Drugs in *Scn1a* ^*E1099X/+*^ Hyperthermia-Induced Seizure Mice

**FIGURE 4:**
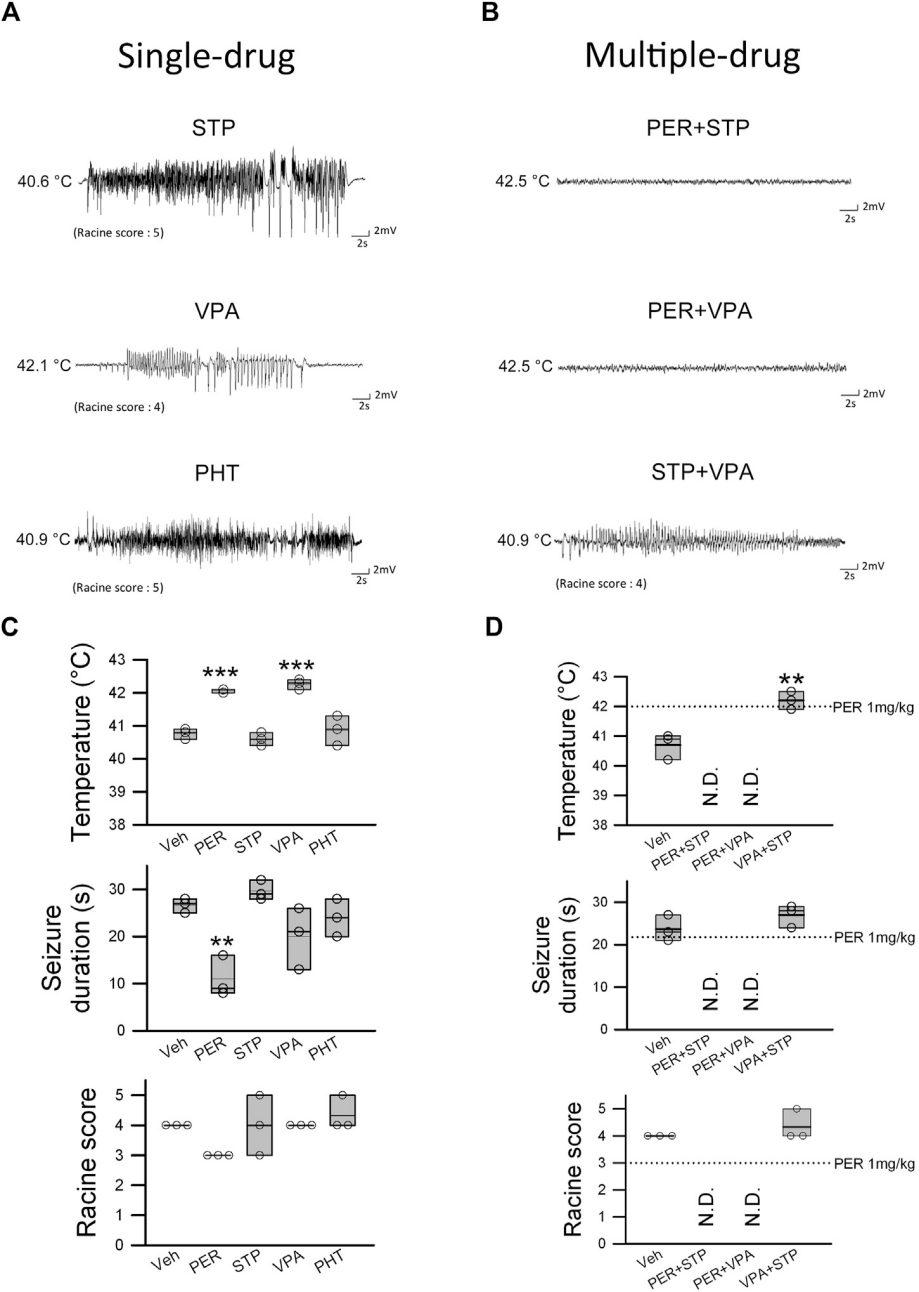
Efficacy of PER in combination therapy for hyperthermia-induced seizure in *Scn1a* ^*E1099X/+*^ mice. **(A)** EEG representative data show the results of single drug STP (stiripentol, 300 mg/kg), VPA (valproic acid, 120 mg/kg), and PHT (phenytoin, 100 mg/kg) treatment. **(B)** EEG images recorded during combination therapy of PER (1 mg/kg) + STP (150 mg/kg), PER (1 mg/kg) + VPA (60 mg/kg), and STP + VPA. The temperature change, seizure duration, and Racine score were recorded for a single drug or combination therapy (*n* = 3 per group). **p < 0.01, ***p < 0.001. **(C)** One-way ANOVA followed by Tukey’s multiple comparisons post hoc analysis in temperature and seizure duration and **(D)** Student’s unpaired *t*-test. N.D., not determined.

To examine the therapeutic effects of other antiepileptic treatments and compare them to PER, we performed the same hyperthermia-induced seizure experiment by giving stiripentol (300 mg/kg), valproic acid (120 mg/kg), and phenytoin (100 mg/kg). Stiripentol and valproic acid are first-line treatments for Dravet syndrome, and phenytoin is one of the most commonly used AEDs clinically. With regard to hyperthermia-induced seizure susceptibility, only valproic acid significantly increased the induced core temperature (average onset temperature: 42.3 ± 0.1°C) compared to the vehicle group (average onset temperature: 40.8 ± 0.1°C) (F_(3,8)_ = 24.470; *p* = 0.0006). In contrast, none of the drugs were found to be beneficial with regard to hyperthermia-induced seizure severity. For both seizure duration and Racine score, there was no significant difference among the groups (Figures 4A,C). Thus, the efficacy of PER among these AEDs is the most prominent.

### Efficacy of PER in Combination Therapy for Hyperthermia-Induced Seizure

Patients with Dravet syndrome are often treated with combination therapy. We examined a few drug combinations using the same hyperthermia-induced seizure experimental protocol. All drugs were given at one-half the dose used in the previous experiment in order to avoid side effects from excessive doses. Therefore, the doses were PER 1 mg/kg, valproic acid 60 mg/kg, and stiripentol 150 mg/kg. In the perampanel + stiripentol and perampanel + valproic acid groups, none of the mice had induced seizure before the temperature reached 42.5°C. In the valproic acid + stiripentol group, the temperature for seizure induction was significantly increased to 42.2 ± 0.2°C compared with that in the vehicle group for seizure induction of 40.7 ± 0.2°C (*t*_(4)_ = 4.910, *p* = 0.008). However, the valproic acid + stiripentol group produced no significant benefit with regard to hyperthermia-induced seizure severity. Seizure duration was 23.7 ± 1.8 s in the vehicle group and 27 ± 1.5 s in the valproic acid + stiripentol group, and the Racine score was 4 in the vehicle group and 4.3 in the valproic acid + stiripentol group (Figures 4B,D). These results showed that PER had high efficacy and a synergistic effect in attenuating heat-induced seizure when combined with stiripentol or valproic acid.

## Discussion

We demonstrated that, in an *Scn1a* ^*E1099X/+*^ transgenic mouse model to simulate Dravet syndrome, PER not only decreased the occurrence of spontaneous recurrent seizures but also when administered in combination with valproic acid or stiripentol had significant therapeutic effects on hyperthermia-induced seizures with regard to both susceptibility and severity. We believe our study is the first to assess the antiepileptic effects of PER combined with first-line AEDs in a Dravet syndrome mouse model, which shows significant therapeutic potential on hyperthermia-induced seizures with regard to both susceptibility and severity.

In our Dravet syndrome mouse model, when PER was given alone, it exhibited antiseizure activity. This is consistent with reports of PER used as monotherapy. An examination of data from a national multicenter registry showed that when PER was administered as monotherapy in patients with focal or generalized tonic–clonic seizures, it was effective at a low dose in most patients (Toledano Delgado et al., 2020). When used as monotherapy, the effectiveness of PER was also reported for the patients with genetic generalized epilepsy (Alsaadi et al., 2019). Furthermore, we found that PER combined with first-line AEDs valproic acid or stiripentol in Dravet syndrome mice is consistent with findings in laboratory and clinical studies. For example, in a recent study, it was demonstrated that the combination of PER (100 nmol/L) and decanoic acid (10 μmol/L) produced a synergistic interaction that directly suppressed seizure-induced activity in human brain slices and in an *ex vivo* model (Augustin et al., 2018). Another study found that stiripentol in combination with clobazam and valproic acid exhibited clinical efficacy in a mouse maximal electroshock–induced seizure (Luszczki et al., 2010). When PER has been used as add-on therapy for idiopathic generalized epilepsy, it is much more effective when given as early add-on therapy than after three or more antiepileptic drugs have been administered (Villanueva et al., 2018).

Patients with Dravet syndrome usually have fever sensitivity throughout their clinical course, and fever could be a seizure-provoking factor (Catarino et al., 2011). Both seizure and ictal activity can be induced or facilitated during fever states, either directly or by high body temperature (typically in the range 38–40°C). Our previous study also demonstrated that the seizure-threshold temperature in *Scn1a* ^*E1099X/+*^ mice was significantly reduced (Tsai et al., 2015). In the present study, we found that both monotherapy and combination therapy with PER significantly increased temperature tolerance (to around 42.5°C) and attenuated seizure occurrence of hyperthermia-induced seizures in the Dravet syndrome mice. Our findings are similar to those in other studies that have reported increases in threshold temperature for seizure onset in Dravet syndrome mice after clobazam alone, stiripentol + clobazam, or stiripentol + clobazam + valproic acid combination therapy (Cao et al., 2012; Hawkins et al., 2017; Pernici et al., 2021).

The mechanism of antiepileptic activity of PER is believed to involve reducing glutamate neurotransmission. One type of glutamate receptor is the AMPA receptor, which is responsible for fast-synaptic transmission in the central nervous system. AMPA receptors are formed as heterotetramers from combinations of different subunits, including GluA1, GluA2, GluA3, and GluA4. Various combinations of subunits are assembled into ion channels with different physiological properties and play an important role in synaptic plasticity (Greger et al., 2017). A major significant distinction among AMPA receptors is between GluA2-containing and GluA2-lacking receptors. GluA2-containing receptors are not permeable to calcium ions, whereas GluA2-lacking receptors are permeable to calcium ions with higher conductivity (Diering and Huganir, 2018). With regard to the expression of AMPA receptor subunits, PER may have the ability to modulate some of the subunits’ expression in a way other than directly inhibiting the AMPA receptor–mediated current. Kim JE et al. (2019) reported that PER regulated the up-stream signal pathway of GluA1, including elevated pCAMKII (Ca^+^–calmodulin–dependent protein kinase II), elevated pPKA (protein kinase A) ratios, and increased pJNK and pPP2B ratios, which results in phosphorylated GluA1-S831 and -S845 to reduce GluA1 protein expression in epileptic rats. These findings indicated that PER might exert antiepileptic effects on blockade of the AMPA receptor and regulate the multiple-molecule–mediated phosphorylation of GluA1 expression.

Compared to some of the most commonly used antiepileptic drugs such as valproic acid, stiripentol, and phenytoin, PER demonstrates better attenuation of hyperthermia-induced seizure. Among these drugs, valproic acid and PER have the ability to decrease heat-induced susceptibility, but only PER can reduce heat-induced severity. We found that phenytoin administration (100 mg/kg) in mice with severe recurrent seizures (Racine score up to 5) 10 min after the first hyperthermia-induced seizure was observed, which showed that a sodium channel blocker may worsen Dravet syndrome and thus should be avoided. Similar to that reported in the previous study, lamotrigine treatment significantly worsened the hyperthermia-induced seizure response (Hawkins et al., 2017). A possible reason is that Dravet syndrome itself is a disease caused by a sodium ion channel defect that leads to the reduction of GABA release (Wirrell and Nabbout, 2019). Stiripentol is a GABA neurotransmission enhancer, which has the ability to enhance GABAergic inhibition and prolong the open duration of GABA-A receptor chloride channels by a barbiturate-like mechanism. We showed that solely enhancing GABA neurotransmission might provide only limited ability to ameliorate heat-induced seizures. Especially when comparing PER (2 mg/kg) and stiripentol (300 mg/kg), we noticed that stiripentol not only had a less therapeutic effect but also had more severe adverse effects on sedation and ataxia during the hyperthermia-induced seizure experiment. Valproic acid has multiple mechanisms; it not only inhibits sodium influx into the cell by the voltage-gated sodium channels but also induces chloride influx by the GABA-mimetic effect. Valproic acid has also been shown to limit the activity of the low-threshold T-type calcium channel. With this effect, valproic acid has the ability to decrease heat-induced susceptibility to seizure, but not severity. Previous studies revealed that seizure severity, including seizure duration, may be related to glutamate transmission and uptake (Xue et al., 1994). A study of resected brain tissue from children with intractable seizures showed that this tissue was hyperexcitable and that PER inhibited the epileptiform activity on the tissue (Wright et al., 2020). This result may be one of the reasons why PER can improve heat-induced seizure severity. In summary, the AMPA receptor may be an effective therapeutic target in Dravet syndrome.

Notably, we found that NBQX had almost the same effect on hyperthermia-induced seizures as perampanel. One of the reasons that NBQX cannot be used clinically is due to its poor solubility. Intravenous NBQX may precipitate into the renal tubules, causing nephrotoxicity (Laurenza et al., 2015). In a clinical study, Laurenza A et al. indicated that patients with partial seizures treated with different doses of perampanel (2, 4, 8, and 12 mg) did not induce liver toxicity (Xue et al., 1994). Therefore, perampanel is currently the one and only AMPA receptor antagonist used clinically. With regard to PER dosage, it is noteworthy that there was no association between the increased levels of PER and central nervous system toxicity (Yamamoto et al., 2018).

There are at least two potential limitations concerning the results of this study. The first limitation concerns the effect of PER on spontaneous recurrent seizures (Figure 1). Dravet syndrome mouse is known to have significant inter-individual variability in spontaneous recurrent seizures. It should be noted that our study is performed only with small sample size and may limit the interpretation of the results. The second limitation is that we used a Dravet syndrome mouse model. There are differences in types of neurons and neuronal properties between rodents and humans. One such example is difference in 5-hydroxytryptamine3 (5-HT3) receptors (Parker et al., 1996). These receptors are common on excitatory neurons in the human forebrain, but in the rodent brain, they are only found on inhibitory GABA neurons.

## Conclusion

Our results illustrate that treatment with PER ameliorated spontaneous recurrent seizures and suppressed epileptic activity in Dravet syndrome mice (*Scn1a* ^*E1099X/+*^ *mice*). In mice with hyperthermia-induced seizures, PER significantly improved the epileptic manifestations and showed synergistic effects when combined with other antiepileptic drugs. The therapeutic potential of PER deserves to be further examined in regard to intractable epilepsy as well as other conditions including neuronal hyperexcitability diseases.

## Data Availability

The original contributions presented in the study are included in the article/Supplementary Material, and further inquiries can be directed to the corresponding author.

## References

[B1] Alsaadi T. Kassie S. Servano R. (2019). Efficacy and Tolerability of Perampanel in Patients with Genetic Generalized Epilepsy (GGE): A Retrospective, Single-center Study from the United Arab Emirates (UAE). Epilepsy Behav. Rep. 12, 100330. 10.1016/j.ebr.2019.100330 31517268 PMC6737327

[B2] Aras L. M. Isla J. Mingorance-Le Meur A. (2015). The European Patient with Dravet Syndrome: Results from a Parent-Reported Survey on Antiepileptic Drug Use in the European Population with Dravet Syndrome. Epilepsy Behav. 44, 104–109. 10.1016/j.yebeh.2014.12.028 25666511

[B3] Augustin K. Williams S. Cunningham M. Devlin A. M. Friedrich M. Jayasekera A. (2018). Perampanel and Decanoic Acid Show Synergistic Action against AMPA Receptors and Seizures. Epilepsia 59, e172–e178. 10.1111/epi.14578 30324610

[B4] Cao D. Ohtani H. Ogiwara I. Ohtani S. Takahashi Y. Yamakawa K. (2012). Efficacy of Stiripentol in Hyperthermia-Induced Seizures in a Mouse Model of Dravet Syndrome. Epilepsia 53, 1140–1145. 10.1111/j.1528-1167.2012.03497.x 22578034

[B5] Catarino C. B. Liu J. Y. W. Liagkouras I. Gibbons V. S. Labrum R. W. Ellis R. (2011). Dravet Syndrome as Epileptic Encephalopathy: Evidence from Long-Term Course and Neuropathology. Brain 134, 2982–3010. 10.1093/brain/awr129 21719429 PMC3187538

[B6] Diering G. H. Huganir R. L. (2018). The AMPA Receptor Code of Synaptic Plasticity. Neuron 100, 314–329. 10.1016/j.neuron.2018.10.018 30359599 PMC6214363

[B7] Dravet C. Oguni H. (2013). Dravet Syndrome (Severe Myoclonic Epilepsy in Infancy). Handb Clin. Neurol. 111, 627–633. 10.1016/B978-0-444-52891-9.00065-8 23622210

[B8] Frey N. Bodmer M. Bircher A. Rüegg S. Jick S. S. Meier C. R. (2017). The Risk of Stevens-Johnson Syndrome and Toxic Epidermal Necrolysis in New Users of Antiepileptic Drugs. Epilepsia 58, 2178–2185. 10.1111/epi.13925 29027197

[B9] Greenwood J. Valdes J. (2016). Perampanel (Fycompa): A Review of Clinical Efficacy and Safety in Epilepsy. P T 41, 683–698. 27904300 PMC5083075

[B10] Greger I. H. Watson J. F. Cull-Candy S. G. (2017). Structural and Functional Architecture of AMPA-type Glutamate Receptors and Their Auxiliary Proteins. Neuron 94, 713–730. 10.1016/j.neuron.2017.04.009 28521126

[B11] Hanada T. Hashizume Y. Tokuhara N. Takenaka O. Kohmura N. Ogasawara A. (2011). Perampanel: a Novel, Orally Active, Noncompetitive AMPA-Receptor Antagonist that Reduces Seizure Activity in Rodent Models of Epilepsy. Epilepsia 52, 1331–1340. 10.1111/j.1528-1167.2011.03109.x 21635236

[B12] Hanada T. Ido K. Kosasa T. (2014). Effect of Perampanel, a Novel AMPA Antagonist, on Benzodiazepine‐resistant Status Epilepticus in a Lithium‐pilocarpine Rat Model. Pharmacol. Res. Perspect. 2, e00063. 10.1002/prp2.63 25505607 PMC4186423

[B13] Hawkins N. A. Anderson L. L. Gertler T. S. Laux L. George A. L. Jr. Kearney J. A. (2017). Screening of Conventional Anticonvulsants in a Genetic Mouse Model of Epilepsy. Ann. Clin. Transl Neurol. 4, 326–339. 10.1002/acn3.413 28491900 PMC5420810

[B14] Kim J.-E. Choi H.-C. Song H.-K. Kang T.-C. (2019). Perampanel Affects Up-Stream Regulatory Signaling Pathways of GluA1 Phosphorylation in Normal and Epileptic Rats. Front. Cel. Neurosci. 13, 80. 10.3389/fncel.2019.00080 PMC6405474 30881292

[B15] Laurenza A. Yang H. Williams B. Zhou S. Ferry J. (2015). Absence of Liver Toxicity in Perampanel-Treated Subjects: Pooled Results from Partial Seizure Phase III Perampanel Clinical Studies. Epilepsy Res. 113, 76–85. 10.1016/j.eplepsyres.2015.03.005 25986193

[B16] Lin K.-L. Lin J.-J. Chou M.-L. Hung P.-C. Hsieh M.-Y. Chou I.-J. (2018). Efficacy and Tolerability of Perampanel in Children and Adolescents with Pharmacoresistant Epilepsy: The First Real-World Evaluation in Asian Pediatric Neurology Clinics. Epilepsy Behav. 85, 188–194. 10.1016/j.yebeh.2018.06.033 30032806

[B17] Luszczki J. J. Trojnar M. K. Ratnaraj N. Patsalos P. N. Czuczwar S. J. (2010). Interactions of Stiripentol with Clobazam and Valproate in the Mouse Maximal Electroshock-Induced Seizure Model. Epilepsy Res. 90, 188–198. 10.1016/j.eplepsyres.2010.04.006 20493662

[B18] Lüttjohann A. Fabene P. F. Van Luijtelaar G. (2009). A Revised Racine's Scale for PTZ-Induced Seizures in Rats. Physiol. Behav. 98, 579–586. 10.1016/j.physbeh.2009.09.005 19772866

[B19] Mantegazza M. Curia G. Biagini G. Ragsdale D. S. Avoli M. (2010). Voltage-gated Sodium Channels as Therapeutic Targets in Epilepsy and Other Neurological Disorders. Lancet Neurol. 9, 413–424. 10.1016/S1474-4422(10)70059-4 20298965

[B20] Marini C. Scheffer I. E. Nabbout R. Suls A. De Jonghe P. Zara F. (2011). The Genetics of Dravet Syndrome. Epilepsia 52 (Suppl. 2), 24–29. 10.1111/j.1528-1167.2011.02997.x 21463275

[B21] Mohammad H. Sekar S. Wei Z. Moien-Afshari F. Taghibiglou C. (2019). Perampanel but Not Amantadine Prevents Behavioral Alterations and Epileptogenesis in Pilocarpine Rat Model of Status Epilepticus. Mol. Neurobiol. 56, 2508–2523. 10.1007/s12035-018-1230-6 30039334

[B22] Oakley J. C. Kalume F. Yu F. H. Scheuer T. Catterall W. A. (2009). Temperature- and Age-dependent Seizures in a Mouse Model of Severe Myoclonic Epilepsy in Infancy. Proc. Natl. Acad. Sci. 106, 3994–3999. 10.1073/pnas.0813330106 19234123 PMC2656193

[B23] Ogiwara I. Miyamoto H. Morita N. Atapour N. Mazaki E. Inoue I. (2007). Nav1.1 Localizes to Axons of Parvalbumin-Positive Inhibitory Interneurons: a Circuit Basis for Epileptic Seizures in Mice Carrying an Scn1a Gene Mutation. J. Neurosci. 27, 5903–5914. 10.1523/JNEUROSCI.5270-06.2007 17537961 PMC6672241

[B24] Parker R. M. C. Barnes J. M. Ge J. Barber P. C. Barnes N. M. (1996). Autoradiographic Distribution of [ 3 H]-(S)-zacopride-labelled 5-HT 3 Receptors in Human Brain. J. Neurol. Sci. 144, 119–127. 10.1016/s0022-510x(96)00211-0 8994113

[B25] Pernici C. D. Mensah J. A. Dahle E. J. Johnson K. J. Handy L. Buxton L. (2021). Development of an Antiseizure Drug Screening Platform for Dravet Syndrome at the NINDS Contract Site for the Epilepsy Therapy Screening Program. Epilepsia 62 (7), 1665–1676. 10.1111/epi.16925 34002394 PMC8360068

[B26] Rosenfeld W. Conry J. Lagae L. Rozentals G. Yang H. Fain R. (2015). Efficacy and Safety of Perampanel in Adolescent Patients with Drug-Resistant Partial Seizures in Three Double-Blind, Placebo-Controlled, Phase III Randomized Clinical Studies and a Combined Extension Study. Eur. J. Paediatric Neurol. 19, 435–445. 10.1016/j.ejpn.2015.02.008 25823975

[B27] Rugg-Gunn F. (2014). Adverse Effects and Safety Profile of Perampanel: a Review of Pooled Data. Epilepsia 55 (Suppl. 1), 13–15. 10.1111/epi.12504 24400692

[B28] Toledano Delgado R. García‐Morales I. Parejo‐Carbonell B. Jiménez‐Huete A. Herrera‐Ramirez D. González‐Hernández A. (2020). Effectiveness and Safety of Perampanel Monotherapy for Focal and Generalized Tonic‐clonic Seizures: Experience from a National Multicenter Registry. Epilepsia 61, 1109–1119. 10.1111/epi.16548 32511754

[B29] Tsai M.-S. Lee M.-L. Chang C.-Y. Fan H.-H. Yu I.-S. Chen Y.-T. (2015). Functional and Structural Deficits of the Dentate Gyrus Network Coincide with Emerging Spontaneous Seizures in an Scn1a Mutant Dravet Syndrome Model during Development. Neurobiol. Dis. 77, 35–48. 10.1016/j.nbd.2015.02.010 25725421

[B30] Villanueva V. Montoya J. Castillo A. Mauri-Llerda J. Á. Giner P. López-González F. J. (2018). Perampanel in Routine Clinical Use in Idiopathic Generalized Epilepsy: The 12-month GENERAL Study. Epilepsia 59, 1740–1752. 10.1111/epi.14522 30062784

[B31] Wirrell E. C. Nabbout R. (2019). Recent Advances in the Drug Treatment of Dravet Syndrome. CNS Drugs 33, 867–881. 10.1007/s40263-019-00666-8 31549357

[B32] Wirrell E. C. (2016). Treatment of Dravet Syndrome. Can. J. Neurol. Sci. 43 (Suppl. 3), S13–S18. 10.1017/cjn.2016.249 27264138

[B33] Wright S. K. Wilson M. A. Walsh R. Lo W. B. Mundil N. Agrawal S. (2020). Abolishing Spontaneous Epileptiform Activity in Human Brain Tissue through AMPA Receptor Inhibition. Ann. Clin. Transl Neurol. 7, 883–890. 10.1002/acn3.51030 32426918 PMC7318092

[B34] Wu Y. W. Sullivan J. Mcdaniel S. S. Meisler M. H. Walsh E. M. Li S. X. (2015). Incidence of Dravet Syndrome in a US Population. Pediatrics 136, e1310–e1315. 10.1542/peds.2015-1807 26438699 PMC4621800

[B35] Xue D. Huang Z.-G. Barnes K. Lesiuk H. J. Smith K. E. Buchan A. M. (1994). Delayed Treatment with AMPA, but Not NMDA, Antagonists Reduces Neocortical Infarction. J. Cereb. Blood Flow Metab. 14, 251–261. 10.1038/jcbfm.1994.32 7509339

[B36] Yamamoto Y. Takahashi Y. Horino A. Usui N. Nishida T. Imai K. (2018). Influence of Inflammation on the Pharmacokinetics of Perampanel. Ther. Drug Monit. 40, 725–729. 10.1097/FTD.0000000000000556 30086086

[B37] Yi P.-L. Lu C.-Y. Jou S.-B. Chang F.-C. (2015). Low-frequency Electroacupuncture Suppresses Focal Epilepsy and Improves Epilepsy-Induced Sleep Disruptions. J. Biomed. Sci. 22, 49. 10.1186/s12929-015-0145-z 26150021 PMC4491875

[B38] Yoshitomi S. Takahashi Y. Yamaguchi T. Imai K. Ishii A. Hirose S. (2019). Efficacy and Tolerability of Perampanel in Pediatric Patients with Dravet Syndrome. Epilepsy Res. 154, 34–38. 10.1016/j.eplepsyres.2019.02.014 31035242

[B39] Yu F. H. Mantegazza M. Westenbroek R. E. Robbins C. A. Kalume F. Burton K. A. (2006). Reduced Sodium Current in GABAergic Interneurons in a Mouse Model of Severe Myoclonic Epilepsy in Infancy. Nat. Neurosci. 9, 1142–1149. 10.1038/nn1754 16921370

[B40] Zeng Q.-Y. Fan T.-T. Zhu P. He R.-Q. Bao Y.-X. Zheng R.-Y. (2015). Comparative Long-Term Effectiveness of a Monotherapy with Five Antiepileptic Drugs for Focal Epilepsy in Adult Patients: A Prospective Cohort Study. PLoS One 10, e0131566. 10.1371/journal.pone.0131566 26147937 PMC4493091

